# The complete plastid genome sequence of *Gleditsia sinensis*, an ancient medicinal tree in China

**DOI:** 10.1080/23802359.2020.1791018

**Published:** 2020-07-15

**Authors:** Ji-Qing Bai, Lei Yang, Su Gao, Wei-Feng Zhu, Lu-Qi Huang

**Affiliations:** a College of Pharmacy, Jiangxi University of Traditional Chinese Medicine, Nanchang, China; b Shaanxi University of Chinese Medicine, Xianyang, China; c National Resource Center for Chinese Materia Medica, China Academy of Chinese Medical Sciences, Beijing, China

**Keywords:** *Gleditsia sinensis*, plastid genome phylogenetic analysis

## Abstract

*Gleditsia sinensis* Lam. (Leguminosae) is an ancient medicinal tree in China. In this study, we first characterized the whole plastid genome sequence using the illumination sequencing technology. The length of the plastid genome was 163,151 bp, including the large single copy (LSC) region of 91,515 bp, the small single copy (SSC) region of 19,250 bp, and two reversed duplicate regions (IR) of each 26,193 bp. The genome of *G. sinensis* contains 130 genes, including 85 protein-coding genes, 37 transfer RNA (tRNA) and 8 ribosomal RNA (rRNA). Phylogenetic analysis showed that *G*. *sinensis* was placed as a sister to the congeneric *G*. *japonica*.

The deciduous tree *Gleditsia sinensis* Lam. (Leguminosae), is widely distributed in China, such as Hebei, Shandong, Henan, Shanxi, Shaanxi, Gansu, Jiangsu and Hunan Provinces. The fruit of *G*. *sinensis* is called “Da-Zao-Jiao” in traditional Chinese medicine and is also a natural raw material for washing products. In addition, the spines of *G*. *sinensis* known as “Zao-Jiao-Ci” in traditional Chinese medicine materials with anti-tumor, anti-inflammatory, anti-allergy, anti-angiogensis and antibacterial activities (Zhang et al. 2020). Here, we performed high-throughput sequencing of the whole platid genome of *G*. *sinensis*.

Fresh leaf tissues of *G*. *sinensis* were collected from Dali county, Shaanxi province, China (N33.700844, E100.005013). The voucher specimens (DL191102002) are preserved in herbarium of Shaanxi University of Chinese Medicine. The total genomic DNA of *G*. *sinensis* was isolated from leaf materials by the modified CTAB method (Doyle and Doyle 1987). Then Illumina pair-read sequences were indexed by the Illumina Hiseq 2500 platform. High quality reads were assembled through the MIRA 4.0.2 program (Chevreux et al. 2004) and MITObim version 1.7 (Hahn et al. 2013). The plastid genome was annotated by GENEIOUS R8 (Fan et al. 2016) and manually adjusted by comparing with the plastid genome homologous gene of MK817503. Finally, the annotated plastid genome of *G*. *sinensis* was submitted to GenBank (accession number: MT483984) and the ring genome was mapped using OGDRAW program (Wang et al. 2019; Gao et al. 2020).

The complete plastid genome of *G*. *sinensis* is a typical square circular molecule with a length of 163,151 bp, consisting of a large single copy (LSC) region of 91,515 bp and a small single copy (SSC) region of 19,250 bp, separated by two reversed repeating regions (IR) of 26,193 bp. The circular genome contains 130 genes, including 85 protein-coding genes, 37 tRNA genes and 8 rRNA genes. Most gene species appear as single copies, while 15 gene species appear as double copies. The total GC content of chloroplast genome of *G*. *sinensis* was 35.6%, while the corresponding values of LSC, SSC, and IR regions were 32.8%, 29.2% and 42.5%, respectively.

Phylogenetic trees were constructed using 17 plastid genomes downloaded from NCBI. We used the MAFFT software (Katoh and Standley 2013) to compare the plastid sequence with the default parameters. Phylogenetic analysis was conducted using the MEGA X (Li et al. 2019) program with a total of 1000 bootstrap repeats. The results showed that *G*. *sinensis* and *G*. *japonica* (Figure 1) are sister species.

**Figure 1.**
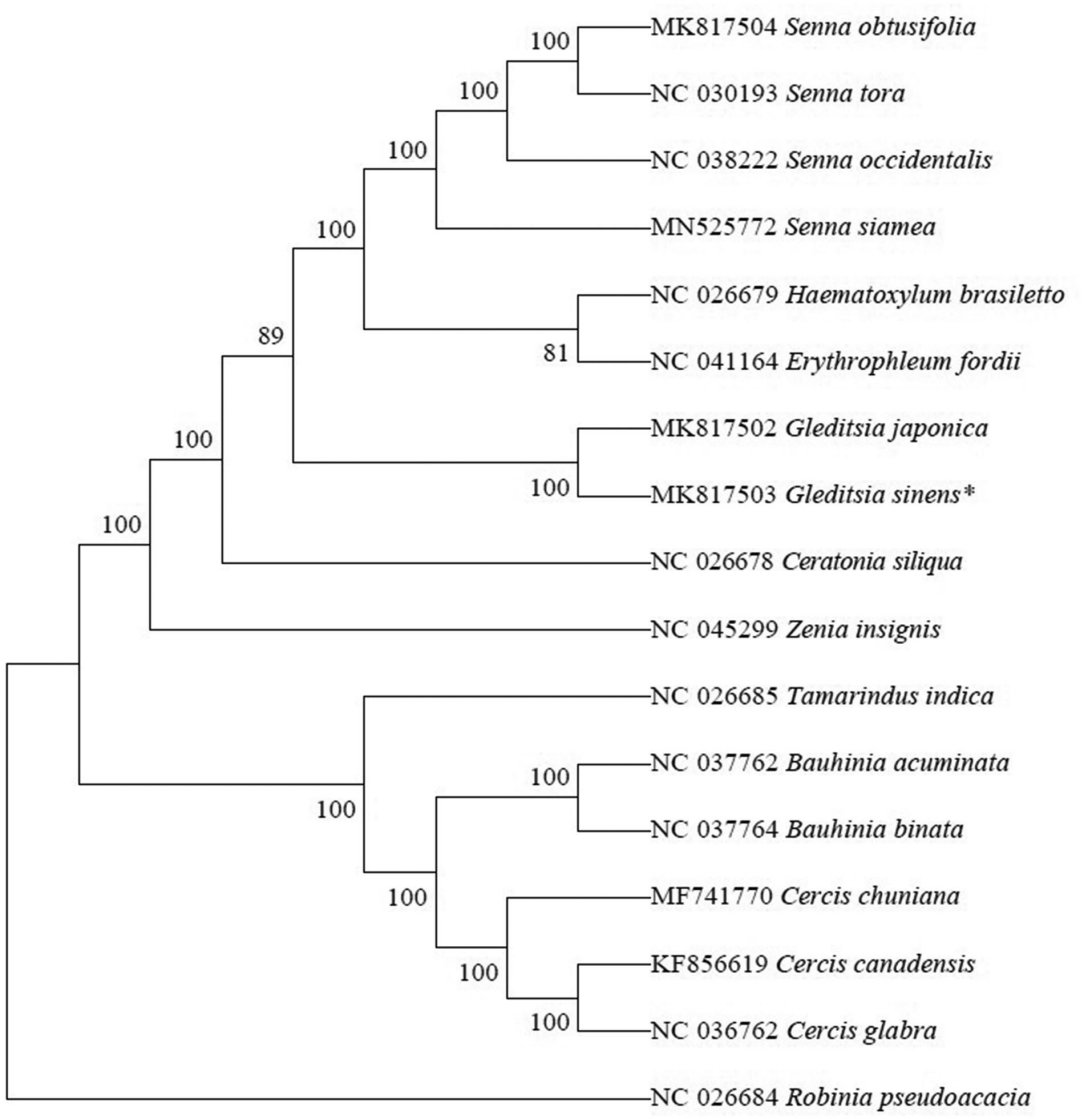
Phylogenetic tree based on 17 complete chloroplast genome sequences. Their accession numbers are before each species names. Numbers in the nodes are the bootstrap values from 100 replicates. * Newly determined chloroplast genomes.

## Data Availability

The data that support the findings of this study are openly available in national center for biotechnology information (NCBI) at https://www.ncbi.nlm.nih.gov/, reference number [MT483984].

## References

[CIT0001] Chevreux B, Pfisterer T, Drescher B, Driesel AJ, Müller WEG, Wetter T, Suhai S. 2004. Using the miraEST assembler for reliable and automated mRNA transcript assembly and SNP detection in sequenced ESTs. Genome Res. 14(6):1147–1159. 15140833 10.1101/gr.1917404 PMC419793

[CIT0002] Doyle JJ, Doyle JL. 1987. A rapid DNA isolation procedure for small quantities of fresh leaf tissue. Phytochem Bulletin. 19(1):11–15.

[CIT0003] Fan K, Sun X-J, Huang M, Wang X-M. 2016. The complete chloroplast genome sequence of the medicinal plant *Rheum palmatum* L. (Polygonaceae). Mitochondrial DNA A DNA Mapp Seq Anal. 27(4):2935–2936. 26153751 10.3109/19401736.2015.1060448

[CIT0004] Gao S, Bai J-Q, Liu M-L, Wang P-F, Li N, Yang L, Wang X-P, Li Z-H. 2020. The complete chloroplast genome of Forsythia mira, an endemic medicinal shrub in China. Mitochondrial DNA Part B. 5(1):57–58. 10.1080/23802359.2019.1693924 PMC7720978 33366420

[CIT0005] Hahn C, Bachmann L, Chevreux B. 2013. Reconstructing mitochondrial genomes directly from genomic next-generation sequencing reads—a baiting and iterative mapping approach. Nucleic Acids Research. 41(13):e129–e129. 23661685 10.1093/nar/gkt371 PMC3711436

[CIT0006] Katoh K, Standley DM. 2013. MAFFT multiple sequence alignment software version 7: improvements in performance and usability. Mol Biol Evol. 30(4):772–780. 23329690 10.1093/molbev/mst010 PMC3603318

[CIT0007] Li Y, Li H, Hei X, Li Y, Li H, Gao J, Yan Y, Liu M, Zhang G. 2019. Characterization of the complete chloroplast genome of medicinal plant Rheum officinale (Polygonaceae). Mitochondrial DNA Part B. 4(2):2144–2145. 33365446 10.1080/23802359.2019.1623102 PMC7687616

[CIT0008] Wang N, Bai J-Q, Dong P-B, Zhang T-T, Wang R-N, Wang J-X, Liu H-Y, Liang R-Y, Tuo P-P, Jing X-T, et al. 2019. Characterization of the complete plastid genome of Cardiocrinum cathayanum, an endemic medicinal plant in China. Mitochondrial DNA Part B. 4(1):1294–1295.

[CIT0009] Zhang YB, Lam KH, Chen LF, et al. 2020. *Gleditsia sinensis* Chemical constituents from the thorns of and their cytotoxic activities. J Asian Nat Prod Res.1–9. 10.1080/10286020.2020.1731799 32290704

